# Three-Electrode, 3D-Printed NMR Cells for Electrooxidation Studies

**DOI:** 10.1021/acs.analchem.5c07786

**Published:** 2026-03-31

**Authors:** Sara A. Salout, Leonid Shupletsov, Irena Senkovska, Arafat H. Khan, Stefan Kaskel, Eike Brunner

**Affiliations:** † Chair of Bioanalytical Chemistry, 9169 Technische Universität Dresden, Bergstraße 66, 01062 Dresden, Germany; ‡ Chair of Inorganic Chemistry I, Technische Universität Dresden, Bergstraße 66, 01062 Dresden, Germany

## Abstract

This study describes a novel experimental apparatus for monitoring electrochemical reactions by in situ NMR spectroscopy. A unique cylindrical 3D-printed electrochemical cell, incorporating a reference electrode, was developed to enable electrochemical measurements through exact potential control. The performance of the novel cell is demonstrated by monitoring ethanol electrooxidation, an important process in fuel cell technology. Ethanol electrooxidation reaction utilizing the UiO-66/Pt/Vulcan XC 72R catalyst was monitored for more than 70 h, providing significant insights into catalyst efficiency, deterioration, and reactivation. This novel methodology fosters the potential of in situ electrochemical investigations, thus widening the applicability in electrocatalysis research.

## Graphical abstract

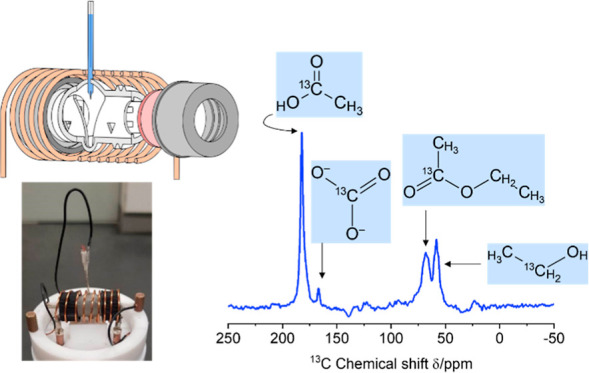

## Introduction

Understanding electrocatalytic reactions at the molecular level is crucial for the development of sustainable energy technologies, such as fuel cells, electrolysis, and redox flow batteries.^1–4^ However, it is challenging to monitor these reactions using traditional characterization techniques because reaction mechanisms may encompass several intermediates and are influenced by surface interactions and the continuously evolving reaction pathway. To address these issues, in situ or operando techniques have become essential for real-time investigations of electrochemical processes.^5–7^ In situ NMR spectroscopy is a unique, powerful technique for investigating electrochemical systems such as batteries and supercapacitors, as well as electrochemical reactions.^8–12^ It provides molecular-level insight into reaction pathway, ion transport, and electrolyte-electrode interactions under operating conditions, while enabling time-resolved, real time tracking of intermediated and interfacial adsorption processes, capabilities that are difficult to access with traditional ex situ methods.^10,13–16^ In situ NMR spectroscopy studies of energy storage devices were pioneered by the group of Clare Grey.^8,10^ Modification of these approaches meanwhile also enabled in situ NMR studies of electrooxidation, especially elucidating the formation of chemical intermediates and catalyst degradation during fuel cell operation.^17–21^ Conventional electrochemical methods, including cyclic voltammetry (CV), chronoamperometry (CA), and electrochemical impedance spectroscopy (EIS), deliver valuable kinetic and mechanistic insights but frequently lack molecular-level specificity. By integrating in situ NMR, researchers may simultaneously monitor the bulk electrolyte and the mobile species adsorbed on the electrode surface. Thus, utilizing this technique, NMR active organic and inorganic species involved in the electrooxidation reaction can be detected, providing insight into the reaction pathway.^5,6,22^

Recently, we reported in situ NMR spectroscopic monitoring of the ethanol electrooxidation reaction (Scheme 1),^9^ which is important for direct ethanol fuel cells (DEFC). The reaction was catalyzed by carbon-supported platinum functionalized with a metal–organic framework (MOF).

**Scheme 1:**
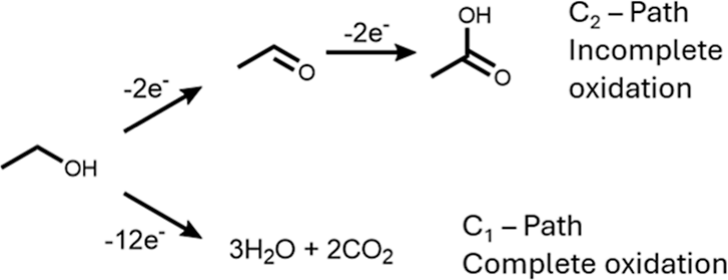
General Scheme of Ethanol Electrooxidation Showing Two Main Pathways: Incomplete Oxidation to Acetic Acid and Aldehyde, and the Complete Oxidation to CO_2_ ^a^

a Balancing with OH- and H_2_O is omitted for Brevity.

Another notable example is provided by Blanc et al. Their work demonstrated that in situ solid-state NMR can detect reaction intermediates such as CO adsorbates and partial oxidation products during methanol oxidation in an operating fuel cell, revealing factors controlling performance and degradation.^10^ Apart from methanol oxidation, Wang et al. applied in situ ^19^F NMR to supercapacitors, identifying distinct charge-storage regimes and tracking ion adsorption/desorption at positive and negative electrodes in real time.^8^ These investigations underscore how in situ NMR can reveal mechanistic details inaccessible to conventional electrochemical measurements.

Although the method provides significant insight, the implementation of the electrochemical cell into an NMR spectrometer poses many challenges which must be addressed first. One possible approach, prior to the development of the novel 3D printed cell-based setup, is described in our previous work by Richter et al.^11^ Here, in situ NMR measurements were conducted in a polyethylene (PE) pouch cell (Figure 1a), which were inserted into the solenoid coil of the NMR probe head (Figure 1b).

**Figure 1:**
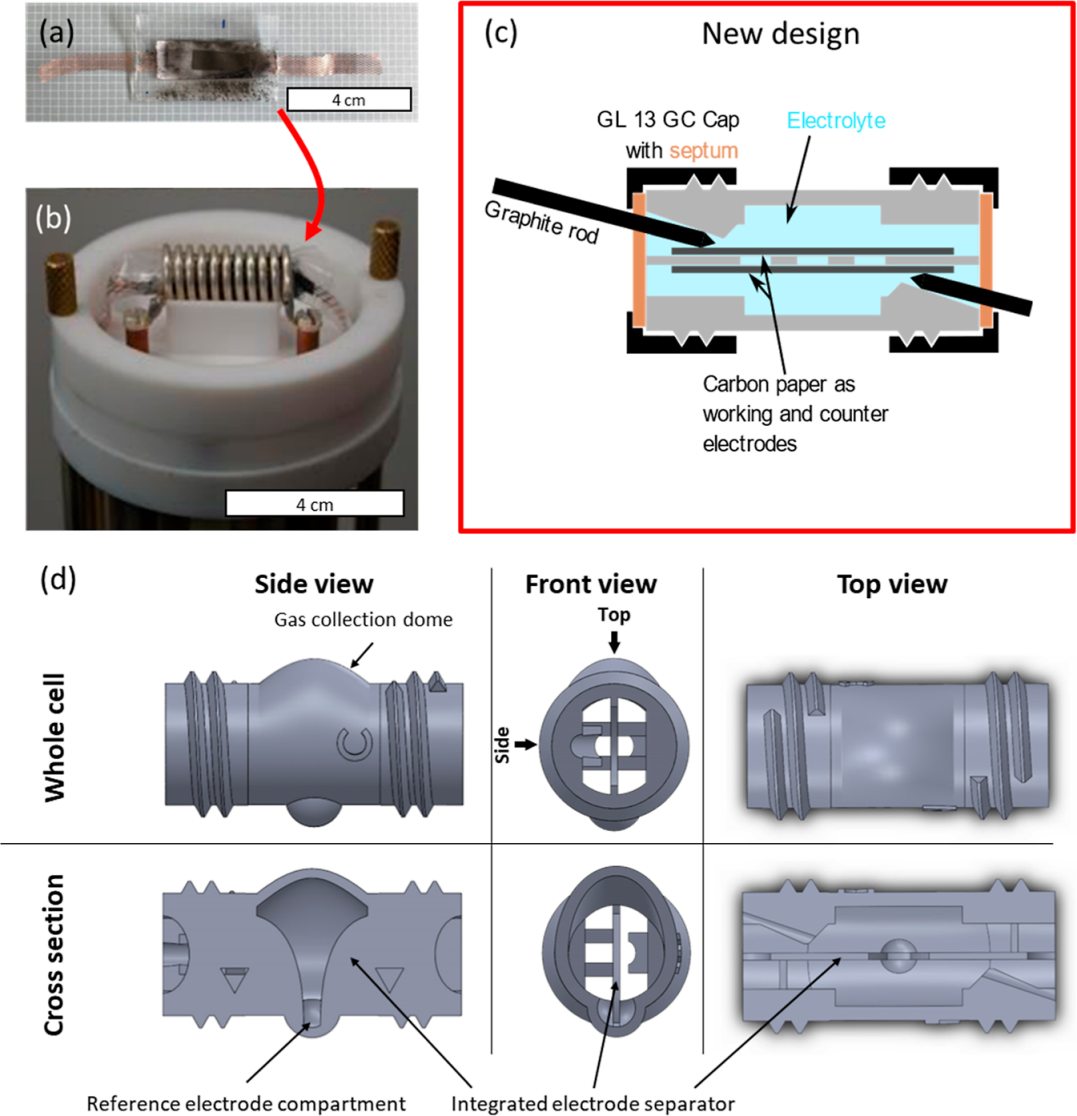
State-of-the-art pouch-cell-based setup before (a) and after (b) insertion into the NMR solenoid coil.^11^ Novel 3D printed cell-based setup for comparison (c,d) side, front, and top views of the novel, 3D cell body model with cross-section views.

The pouch cells typically contained two coated carbon paper electrodes separated by a glass-fiber separator. The carbon papers were contacted by metal meshes, usually made of copper. The electrode assembly was sealed inside a laminated PE pouch that contained the desired electrolyte. Despite its efficacy, several disadvantages of this setup should be mentioned.

The pouch cells are sealed using a heat-sealing machine and are prone to leakage if the seal zone is contaminated by catalyst particles or other solid residues during sealing, as such contamination can hinder uniform melting and fusion of the laminate layers and lead to localized seal defects. The metal electrical contact, which must be integrated and welded into the heat-sealed seam, further introduces an inherent risk of improper sealing by locally disrupting pressure and temperature uniformity at the seal interface.

The flexibility of the pouch cells prevents the setup from maintaining a fixed geometry. This can lead to deformation of the cell and friction of the internal components, which in turn causes detachment of the catalyst coating from the carbon paper or other potential conductive substrate. Furthermore, cell deformation may lead to a loss of contact or short circuits. The presence of the metal mesh as a contacting material may, if the wrong mesh material is chosen, lead to undesired side reactions on it. This can either falsify catalyst performance or cause the loss of contact due to the corrosion of the metal mesh. The metal mesh can also affect the NMR measurement. Manual assembly of pouch cells is detrimental for reproducibility, as every cell may differ in dimensions, contact resistances, electrolyte volume, etc. The lack of a reference electrode (RE) in the setup does not allow for the application of a defined potential, rendering catalytic studies a guessing game about the true potentials on the electrodes. Finally, the pouch cell must be completely sealed during assembly, which does not allow for studying the reactions that generate a gaseous product, as gas accumulation could lead to reaction inhibition and even cell bursting. This problem can be mitigated to a certain extent by puncturing the cell after insertion into the NMR solenoid coil; however, this may result in leakage and cause damage to the probe head.

These challenges are well-known and have been encountered by other researchers, and some solutions have been proposed.^23–29^ However, most of the suggested setups require cost-intensive investments in the necessary equipment. In our opinion, very promising results in this field have so far been demonstrated by Ferreira Da Silva et al.,^24–26^ who miniaturized the three electrodes to a millimeter scale, allowing their incorporation into an NMR tube of 5 mm in diameter. The small size of the electrodes imposes a limitation on the maximum currents achievable in the electrochemical setup, thereby slowing the progress of the reaction. Such electrodes, however, are not commercially available and were fabricated by the researchers as custom-made components. This delicate and skill-intensive process, combined with the fragility of the electrodes and the risk of a short circuit inside the tube, due to the lack of a physical separator between them, is, however, an inherent problem of this approach.

The fabrication of electrochemical cells by 3D printing has become a popular approach in recent years.^30–33^ The versatility of 3D printing further allowed to develop novel in situ measurement setups to combine electrochemistry with spectroscopy methods, for example, with Raman, UV–vis, or X-ray spectroscopy.^34–40^ Similar innovations for NMR spectroscopy have not yet been developed. To address the aforementioned issues, we designed a novel cylindrical 3D printed electrochemical cell for in situ NMR spectroscopy (Figure 1c,d).^41^ In contrast to the previously employed pouch cell setup, the novel cell possesses a rigid geometry, allows for the incorporation of a reference electrode, contains an integrated separator, avoids metal components inside the reaction and NMR detection volume, provides reproducible and safe hermetic sealing, handles reactions with gaseous products, and is affordable and reusable. The proposed design allows for rapid and cost-effective production of the cell bodies via fused filament fabrication (FFF), alleviating the necessity for costly components. To evaluate and optimize the performance of this novel setup, we employed the ethanol electrooxidation reaction again (Scheme 1). As in our preliminary studies, UiO-66/Pt/Vulcan XC 72R was chosen as the catalyst. The in situ NMR measurements were performed in combination with cyclic voltammetry (CV) and chronoamperometry (CA).

## Experimental Section

### Materials

All solvents for synthesis and electrochemical analysis were used without further purification. *N,N*-dimethylformamide (DMF, > 99.5%), ethanol (>99.5%), and HCL (37% aq.) were purchased from Fluka. ZrCl_4_ (>99.5%) was purchased from Thermo Scientific, and 1,4-benzenedicarboxylic acid (H_2_bdc, > 99%) from Acros Organics. ^13^C-2 ethanol was obtained from Sigma-Aldrich (99 atom % ^13^C).

### Synthesis of UiO-66@Pt/C

The catalyst material was prepared according to the protocol developed in our previous work.^42^ Commercial Pt on Vulcan XC72 (10 wt % Pt, Sigma-Aldrich, Lot MKBJ0568 V) (80 mg) was dried in dynamic vacuum at room temperature (23 °C) overnight in a Schlenk tube. It was then added to a solution of terephthalic acid (H_2_bdc) (9.5 mg, 57.4 μmol, 1.4 equiv) in 8.5 mL DMF, and the mixture was stirred overnight at 80 °C under argon flow with a magnetic stirrer inside a round-bottom flask. Subsequently, a solution of ZrCl_4_ (9.4 mg, 41.2 μmol, 1.0 equiv) in 3 mL DMF was added to the reaction mixture under stirring. Finally, HCl (0.8 mL, 37%) was added at once, and stirring ceased. The reaction mixture was sealed under argon and maintained at 80 °C overnight. The resulting fine black powder was separated from the mother liquor by centrifugation and washed with DMF (3 × 7 mL) and EtOH (3 × 7 mL) before drying in a vacuum at 150 °C overnight (16 h). The isolated yield of UiO-66@Pt/C was only 83 mg after vacuum drying at 150 °C, due to significant material loss during washing caused by the very small particle size. The UiO-66 formation was confirmed by powder X-ray diffraction (PXRD) (Figure S1). Only the first and most intensive reflection at 2Θ = 7.4° could be observed, due to the low mass loading and very small crystal sizes, in full accordance with our previous work.^42^

### Catalyst Preparation

The catalyst-coated carbon was prepared by first ball milling UiO-66@Pt/C (50 mg) in a water/ethanol mixture (1:1; 1 mL) for 5 min in a vibratory ball mill (Vibrator, M. v. Ardenne) at 10 Hz equipped with an 8.2 mL stainless steel vessel and a 4 g stainless steel milling ball. The obtained slurry was doctor-bladed with a blade height of 1250 μm on a 20 × 60 mm^2^ AvCarb P50 carbon paper (paper thickness 200 μm) at a blade speed of 100 mm s^–1^. The resulting paper was dried in air for 2 h first, before drying at 80 °C in a dynamic vacuum overnight. The coated paper was stored in air and reactivated at 80 °C in a dynamic vacuum for 2 h prior to use. The papers were weighed before and after coating to assess the catalyst amount. Strips of 20 × 6 mm^2^ were cut from the coated paper in air and used in the electrochemical experiments. Under the assumption of uniform coating, the mass of the UiO-66@Pt/C composite per strip is 3.4 mg. This corresponds to 320 μg of platinum per strip.

### Electrochemical Measurements

All electrochemical experiments were conducted with the catalyst-coated carbon paper as the working electrode (WE) and a noncoated carbon paper of the same geometry and area as the counter electrode (CE). A reference electrode (RE) was utilized as stated in the individual experiments. The total volume of the electrolyte in the cell was always 0.8 mL. Cyclic voltammetry (CV) experiments were conducted with a scan rate (*v*) of 50 mV s^–1^.

The in situ electrooxidation experiments were conducted with an optimized potential sequence (Figure 2).

**Figure 2:**
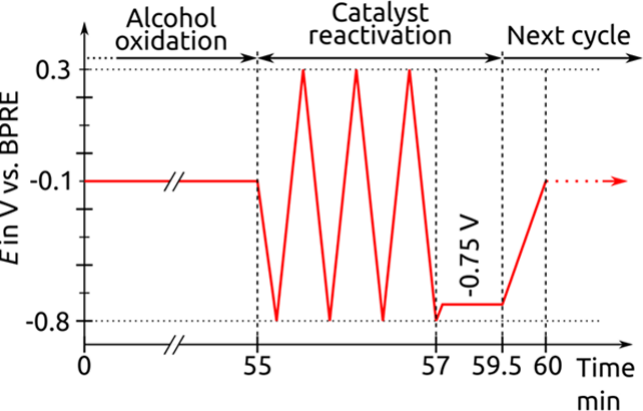
Applied potential sequence during in situ electrooxidation NMR experiments.

First, a potential of −0.1 V vs bipolar reference electrode (BPRE) was applied for 55 min during the chronoamperometric (CA) step of the ethanol electrooxidation. Following this initial oxidation step, a cyclic potential range from −0.8 to 0.3 V (*v* = 50 mV s^–1^) was applied for three cycles to reactivate the electrode surface. Finally, a reducing potential of −0.75 V was applied for 150 s followed by repeating the sequence. During electrochemical reactions, impurities from the electrolyte, reactants, or the environment can adsorb onto the electrode surface, forming a layer that inhibits catalytic activity.^25^ Additionally, reaction products or intermediates, e.g. CO may adsorb on the electrode surface, blocking active sites and retarding the electrode or catalyst efficiency. The described potential cycling effectively removes adsorbed species and other impurities from the catalyst surface, while the reducing potential reduces the Pt nanoparticles to Pt^0^, thus reactivating them for the next oxidation cycle. One whole sequence lasted exactly 1 h and was repeated 80 times during the in situ NMR measurements.

### In Situ NMR Spectroscopy

In situ NMR spectroscopic studies were conducted using a 300 MHz Bruker AVANCE spectrometer. A specialized probe, manufactured by NMR Service GmbH (Erfurt, Germany, version of 2015, single channel detection of X nuclei), was utilized for investigating the novel cell designs. A handmade RF solenoid NMR coil with a 15 mm diameter and 3 cm length was prepared to fully enclose the plastic cell with a moderate *Q* factor and an optimal filling factor with the help of Rigol DSA815 spectrum analyzer (LXI, 9 kHz—1.5 GHz). Electrical connection of the cell electrodes to the probe was implemented using stranded PVC-insulated copper wires with a conductor cross section of 0.14 mm^2^. The stranded wire was chosen because it provided the best contact with the graphite rods and probe head. A distance of approximately 2 cm was maintained between the cell position inside the RF coil and the probe cap, providing sufficient space for insertion and positioning of the reference electrode.

To minimize noise during in situ NMR measurements, SLP-15+ low-pass filters (DC-15 MHz, 50 Ω) manufactured by Mini-Circuits were installed between the electrodes and the potentiostat, preventing interference of NMR pulses with potentiometric measurements. The electrochemical investigations employed an IviumStat potentiostat from Ivium Technologies. In situ ^13^C NMR spectra were acquired using direct, single pulse (SP) excitation using a 45° flip angle (11.25 μs duration at PL1:1.50 dB corresponding to half of the calibrated 90° pulse; 25 kHz spectral width), selected to optimize signal-to-noise per unit time under partially relaxed conditions. Each spectrum consisted of 900 individual scans, with a recycle delay of 4 s applied between successive scans. Proton decoupling was not applied due to the use of a single channel in situ probe tuned exclusively to ^13^C. As the material properties of the cell components may change during the electrochemical reactions, altering the magnetic susceptibility of the detected substances, recalibration (tuning and matching) was performed daily. The chronoamperometric voltage and current for each cell measurement were recorded to monitor the stability of the electrochemical cell during the measurements.

High-resolution transmission electron microscopy (HR-TEM) and scanning transmission electron microscopy with energy-dispersive X-ray spectroscopy (STEM-EDX) were also applied.

HR-TEM images were taken with a JEOL Jem F-200C transmission electron microscope equipped with a Gatan OneView—in situ 4K camera at an acceleration voltage of 200 kV. The STEM-EDX measurements were conducted with the same device and acceleration voltage, utilizing a 100 mm^2^ JEOL windowless Dual EDX system. The convergence angle was set to 10 mrad. The samples were drop-cast on TED Pella ultrathin carbon film on lacey carbon support 400 mesh copper grids from ethanol prior to the measurement.

### 3D Modeling and Cell Printing

3D models of all 3D printed parts were created with the SolidWorks 2023 (Dassault Systèmes) software package.^43^ The models were transformed into STL format with high precision and sliced with the Cura 5.0.3 (UltiMaker) slicer program.^44^ The prints were executed on a Creality CR-5 Pro fused filament fabrication (FFF) printer equipped with a 0.4 mm nozzle using transparent polypropylene (PP) filament from Formfutura. The printing temperature was 225 °C. Tightness of the prints was tested by filling the cell bodies with 1 mL of EtOH abs. and sealing them with ND13 screw caps with PTFE/silicone laminate septa (PTFE layer facing the sample for chemical resistance, silicone layer providing self-sealing elasticity). The assembled cells were kept at room temperature with no agitation. The total mass of each cell was monitored over several days.

## Results and Discussion

### Novel Cell Design

Previous electrochemical in situ NMR cells exhibited several limitations as discussed above. Due to their flexibility and lack of structural stability, pouch cells typically require manual welding, in which the metal contacts are inserted and fused into the seal with a vacuum pouch cell welding machine. This delicate process results in reproducibility issues and an increased risk of leakage due to the aforementioned reasons (e.g., contamination of the seam, poor polymer-to-metal adhesion) (Figure 1a). Additionally, the use of metal wires for electrode contacts in pouch cells can cause undesirable side reactions and even degrade over time at harsh pH conditions. The soft cell structure renders it difficult to install a reference electrode, which is crucial for applying stable electric potentials. Solid cells made of polytetrafluoroethylene (PTFE) or glass are expensive, difficult to modify, and often lack reference electrodes, thus limiting their suitability for various electrochemical studies.^28,29,45^ To address these issues, we developed a novel cylindrical 3D-printed cell body with integrated separators, which are hermetically sealable by commercially available ND13 screw caps with PTFE/silicone laminate septa (Figure 1c). This design allows the insertion of thin graphite electrodes and ensures metal-free electrode contact, reducing interference signals in NMR measurements.

The cell body is easily adjustable and manufacturable on-site using 3D printing, allowing customization to meet experimental requirements, such as internal volume, electrode spacing, port positions, and overall geometry. This new cell design is cost-effective, with all components being inexpensive and readily available. The setup process is quick and requires minimal training. The cell body is reusable, while only the septa and graphite electrodes need to be replaced after every experiment. The design supports reactions involving gases and can function independently as a measurement setup for electrochemical investigations, with potential adaptability as a flow cell. The cell body incorporates a dome-shaped extrusion that collects evolving gases. These gases are released in a controlled manner through a small perforation at the top of the dome, coinciding with the insertion point of the reference electrode, which allows gas to escape while retaining the liquid electrolyte (Figure 1d). While a similar gas venting functionality could in principle be achieved using conventional machining, the fabrication of such enclosed, curved internal geometries and integrated features would require multistep machining or assembly, significantly increasing fabrication complexity and cost. In contrast, additive manufacturing enables straightforward, single-step fabrication of these internal structures with high reproducibility and design flexibility.

The working and counter electrodes are inserted on either side of the integrated separator, and the ends are sealed with screw caps. The cell is filled with the desired electrolyte via a syringe through one of the septa. Graphite pins are used to contact the electrodes through the septa, pressed firmly by the cell geometry, with one end connecting to the electrode and the other end soldered to stranded PVC-insulated copper wire (0.14 mm^2^ conductor cross section) using heat-shrink tubing to ensure a stable electrical connection. As such, the cell can be introduced into the solenoid coil of the NMR (Figure 3a). The RE can be subsequently inserted through an opening at the top of the cell and positioned in a small spherical cavity at the bottom, if required (Figure 3b). This prevents movement of the RE, and thus, the RE maintains its position strictly between the working and counter electrodes. The cell design also allows for the integration of liquid or gas flow through the dome perforation and septa ports, making it versatile for various electrochemical studies and ensuring stable, reproducible conditions.

**Figure 3:**
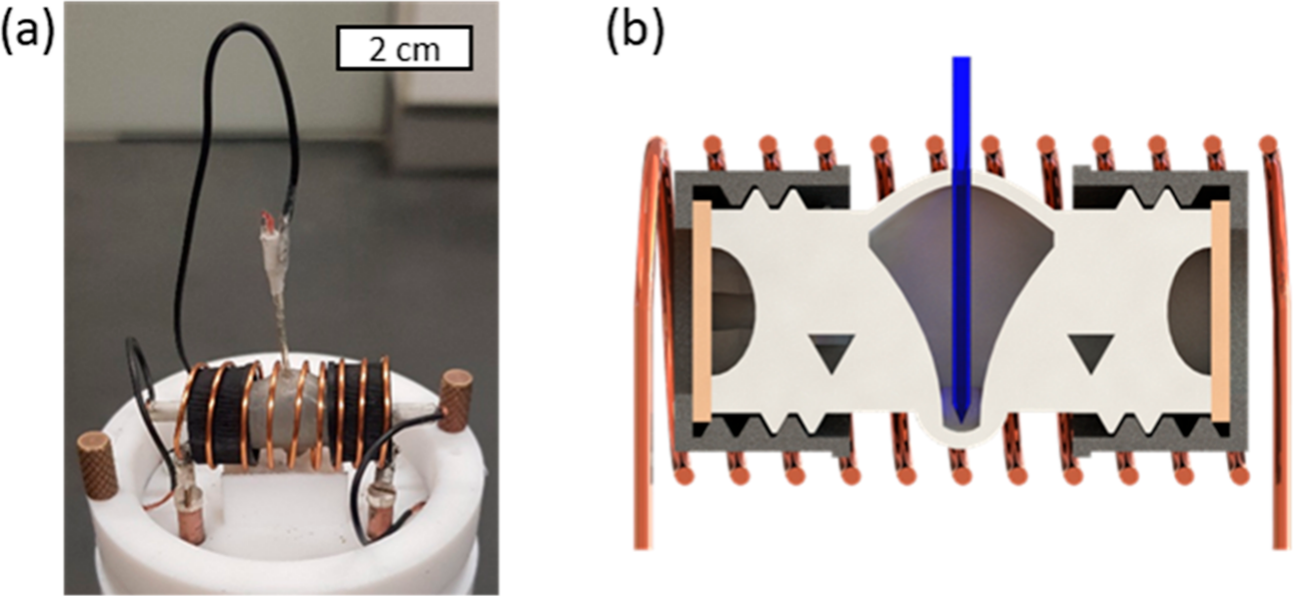
(a) Image of electrochemical setup equipped with the micro BPRE inside the NMR probe head; (b) visualization of the BPRE cross-section positioned inside the measurement cell.

As the FFF printing process is prone to interlayer defects arising from irregularities in the filament, varying moisture levels during printing, and exposure to dust or other particulate matter, which may prevent sufficient layer adhesion, the quality of the printed cell bodies is tested prior to use. For that reason, freshly printed cells were filled with EtOH and sealed with the corresponding screw caps. The mass of the assembled cells was monitored over 9 days. As shown in Figure S2, two out of three manufactured cells demonstrate adequate tightness with a mass loss of only 0.14 and 0.28 wt % per day, respectively. One cell, however, demonstrates substantial ethanol loss from within, amounting to 3.35 wt % per day. This indicates the presence of a layer defect in the print, which renders the given cell unusable. The success rate of the prints highly depends on the quality of the material used, the 3D printer, and the chosen settings. After optimization we achieved a success rate of 80%. Thus, it is imperative to check printed cells before use and utilize only the best-performing cells in subsequent experiments.

### Closed Bipolar Reference Electrode

The leakless BPRE was manufactured according to Walker and Dick,^46^ by sealing a graphite rod (0.5 mm in diameter, 1 cm in length) inside a borosilicate glass capillary tube (1.0 mm outer diameter, 0.7 mm inner diameter, softening point = 821 °C) using a Bunsen burner. The graphite rod was inserted so that at least 1 mm extended beyond the tip of the capillary tube. The end of the tube containing the graphite rod was heated in the flame until it became molten for at least 10 s, ensuring a strong and reproducible seal. After allowing the capillary to cool to room temperature, it was filled with 1 mol L^–1^ KCl. A silver wire, previously anodized in HCl (*c* = 2 mol L^–1^), was then inserted into the back end of the tube. A copper wire was soldered to the anodized silver wire for contacting. The back end of the BPRE was sealed with UV-resin to prevent evaporation (Figure S3a,b). The electrode was then soaked in an electrolyte of the same composition as its internal solution for at least 48 h before validation. Prior to measurements, the function of the BPRE was validated in NaOH solution (*c* = 0.5 mol L^–1^) versus a commercial Hg/HgO electrode. The outstanding stability of the manufactured BPREs can be seen in Figure S3c. Both BPREs used for this study demonstrated the same potential vs. a commercial Hg/HgO electrode and fluctuated by less than 5 mV in their potential (Figure S3c). A second important characteristic of reference electrodes is their impedance. This was measured in a three-electrode setup with a Pt wire as counter electrode, an Ag/AgCl (*c* = 3 mol L^–1^) electrode as reference, and the BPRE as working electrode. With a series resistance of less than 200 Ω and an impedance of less than 60 Ω in the high frequency regime, the BPREs are well suited to be used as reference electrodes (Figure S3d).

### Cell Characterization and Stability Test

To evaluate the compatibility of the new cell design with ^13^C NMR spectroscopy, we conducted single pulse (SP) NMR experiments using the polymer cell body filled with KOH (1 mol L^–1^) (Figure S4). This revealed two broad signals centered at 26 and 133 ppm, originating from the polypropylene of the cell body. A reference spectrum including the polypropylene signals was also measured for a cell filled with KOH (1 mol L^–1^) electrolyte under the same conditions as the ^13^C SP NMR experiment on the reaction solution. This reference spectrum was then subtracted from each spectrum obtained during the in situ NMR experiment to remove the contributions of the cell body.

Next, we assessed the performance of the perforated cell using an electrolyte containing 0.8 mL of KOH (1 mol L^–1^) and ethanol (^13^C_2_-labeled, 1 mol L^–1^) by monitoring the ethanol losses due to evaporation over 70 h without applying any potential (Figure S5a,b). Cell perforation is necessary to accommodate the reference electrode and gas release during the electrooxidation reaction in in situ experiment. Without adequate venting, gas accumulation leads to electrode deactivation.^11^ This setup is identical to that used in in situ experiments (three-electrode system). Our findings indicated a 30% loss of ethanol after 70 h due to perforation. The steady ethanol loss due to evaporation is unfortunately unavoidable in the perforated cell. So far, no effective method has been found to vent evolving gases without losing ethanol or other volatile compounds. No such caveats occur when studying less volatile compounds, such as glycerol electrooxidation (GOR). The potential dependent product distribution and mechanistic insights into GOR were obtained with the help of the setup presented here and reported in our recently published work.^47^ The reproducibility of the measurements is guaranteed by the precision of the cell manufacturing, allowing for benchmarking the evaporation rate in advance and accounting for it in the experiments. This problem was also evident in previous pouch cell experiments. However, the inconsistency of the pouch cells led to varying rates of evaporation in each conducted experiment.^9^ It should be noted that bulk magnetic susceptibility (BMS) effects must be taken into account for operando/in situ NMR spectroscopy studies in general.^8,48–50^ BMS-induced signal shifts can be corrected primarily by filling the sample cell with a reference solution of known chemical shifts, e.g., an ethanol-containing electrolyte (see Figures S4 and S5). However, BMS effects can also give rise to substantial line broadening, which may explain the relatively large full width at half-maximum of approximately 7 to 10 ppm observed for ethanol in solution here. Therefore, experiments were carried out detecting ^13^C with its relatively wide chemical shift range between ca. 0 and 200 ppm. Using the more sensitive ^1^H nucleus with its relatively narrow chemical shift range between ca. 0 and 20 ppm would require a better resolution. Future work may target cell design optimization to minimize BMS effects in order to make in situ ^1^H NMR spectroscopy of electrochemical reactions feasible. This would also allow the use of reactants without cost-intensive isotope enrichment.

### Electrochemical Performance Test

A crucial improvement of the proposed measurement setup is the possibility of including a reference electrode (Figure 3). The necessity of a proper RE becomes evident if comparing the new data to that obtained in a two-electrode setup (I; Figure S6a) and a three-electrode setup with a pseudo reference electrode (II; Figure S6b). The characteristic CV curve of alcohol electrooxidation in alkaline medium contains four main features: **1** – alcohol electrooxidation; **2** – alcohol depletion at the catalyst and catalyst deactivation due to oxidation and poisoning with CO;^51–56^ **3** – catalyst reactivation, e.g., reduction of platinum oxide to metallic platinum and stripping of CO adsorbed; **4** – oxidation of adsorbed species, e.g., alkoxide, on the reactivated Pt nanoparticles (ethanol electrooxidation on pristine Pt/Vulcan catalyst in alkaline conditions is depicted as example in Figure S6d). The two-electrode setup has proven entirely inappropriate for obtaining the characteristic CV shape of the ethanol electrooxidation (Figure S6a). The Pt pseudo reference is sufficient for short experiment times, as it provides an adequately stable potential on a time scale of minutes (Figure S6b). Despite its drawbacks, the Pt pseudo reference demonstrates that the characteristic CV features of ethanol oxidation **1** and **3** are located exactly above (0.10 V vs Pt/Pt^+^) and below (−0.08 V vs Pt/Pt^+^) the platinum redox potential. Thus, demonstrating again that the drop in the oxidation current in the CV (Feature **2** in Figure S6d) is not only caused by diffusion limitation, but also by the oxidation and thus deactivation of the Pt nanoparticles.^57^ Meanwhile, at potentials below the platinum oxidation (Feature **3** in Figure S6d), the reactivation of the nanoparticles occurs, as expected, due to the nanoparticle’s reduction. This leads to increased alcohol oxidation (Feature 4 in Figure S6d), as evidenced by an increase in oxidative current. However, for longer chronoamperometric measurements, the utilization of a pseudoreference is deemed inadequate, as the pseudoelectrode potential drifts over time, especially in a changing electrochemical environment.

In contrast, the BPRE provides a known, stable potential on a time scale of days, allowing the application of well-defined electric potentials to the catalyst (Figure S6c).

### In Situ NMR Spectroscopy Study of Ethanol Electrooxidation

The performance of the above-described new NMR cell was tested in the ethanol electrooxidation reaction (Figure 4). The WE of the cell contained UiO-66@Pt/Vulcan XC 72R catalyst, the CE made from carbon paper AvCarb P50, and BPRE (cf. Figure S3), and the electrolyte containing 0.8 mL of a (1:1 v/v) mixture of 1 mol L^–1^ KOH and 1 mol L^–1 13^C-labeled ethanol, prepared by combining equal volumes of each stock solution. Before conducting in situ NMR measurement of the ethanol oxidation reaction, a CV was recorded from −0.8 to 0.5 V to evaluate cell performance and enable real-time in situ experiment (Figure 4a). The CV recorded inside the NMR coil differs from the CV obtained outside the NMR probe head (Figure S6) because of additional Ohmic resistances and impedances, which are added to all three electrodes, working, counter, and reference, due to necessary circuitry. To avoid interference between the electrochemical setup and the NMR, low-pass filters (DC-15 MHz, 50 Ω) must be inserted between the electrochemical cell and the potentiostat, which distorts the electrochemical measurement. For the potentiostatic oxidation experiment, chronoamperometry (CA) was performed at −0.1 V vs BPRE, since this potential is above the alcohol oxidation potential, but below the oxidation potential of the Pt nanoparticles (see Figure 4a). Thus, the deactivation of the catalyst can be slowed. The potential sequence as described in Figure 2 was applied over 70 h to perform the oxidative catalysis and ensure catalyst reactivation. The first 2.5 h of the sequence are depicted in Figure 4b. The ethanol electrooxidation reaction was monitored in situ by ^13^C SP NMR to track ethanol consumption and product formation (Figure 5) during the entire electrooxidation experiment. The ethanol signal was detected at a chemical shift of 58 ppm. The intensity of this ethanol signal continuously decreases over the measurement time (Figure 5c,d).

**Figure 4:**
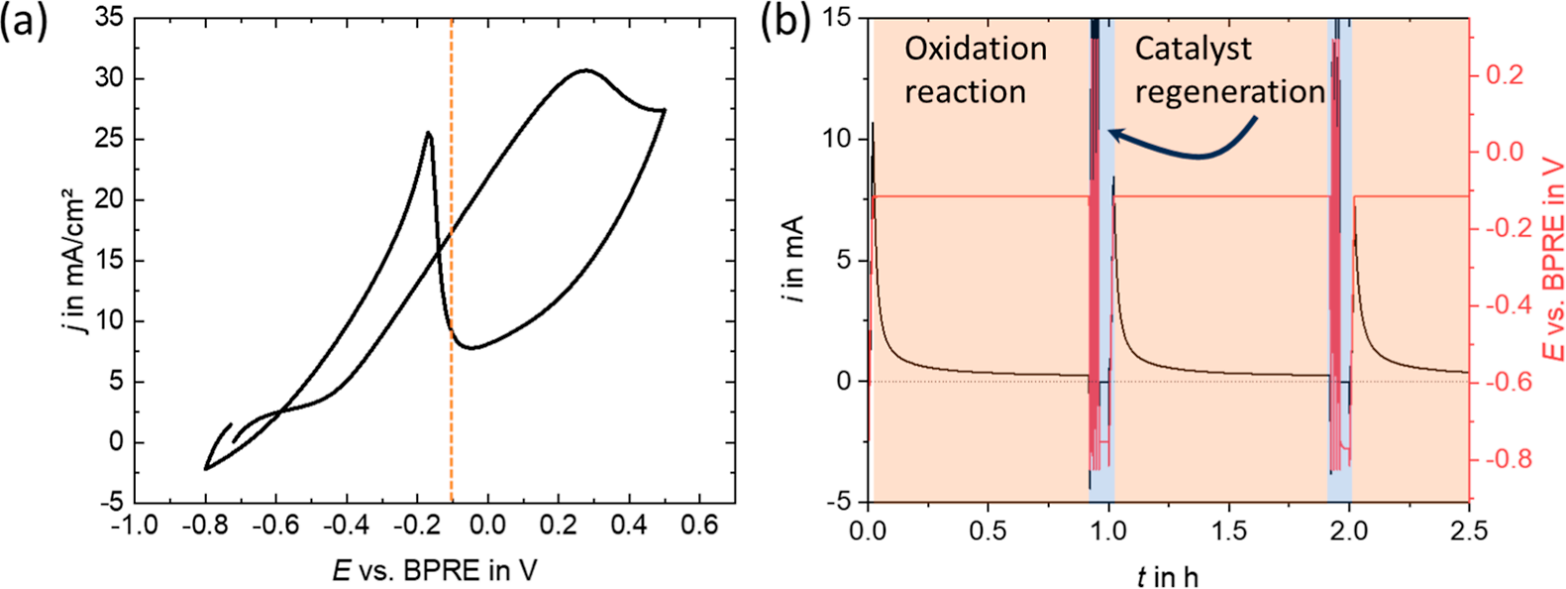
(a) CV of UiO-66/Pt/Vulcan XC 72R catalyst ranging from −0.8 to 0.5 V vs BPRE, collected in 0.8 mL of a (1:1 v/v) ^13^C-labled ethanol and KOH (each 1 mol L^–1^) inside the NMR spectrometer, with chosen electro oxidation potential highlighted; (b) first 2.5 h of in situ electro oxidation experiment of ethanol with UiO-66@Pt/Vulcan XC 72R with oxidation and catalyst reactivation highlighted.

**Figure 5:**
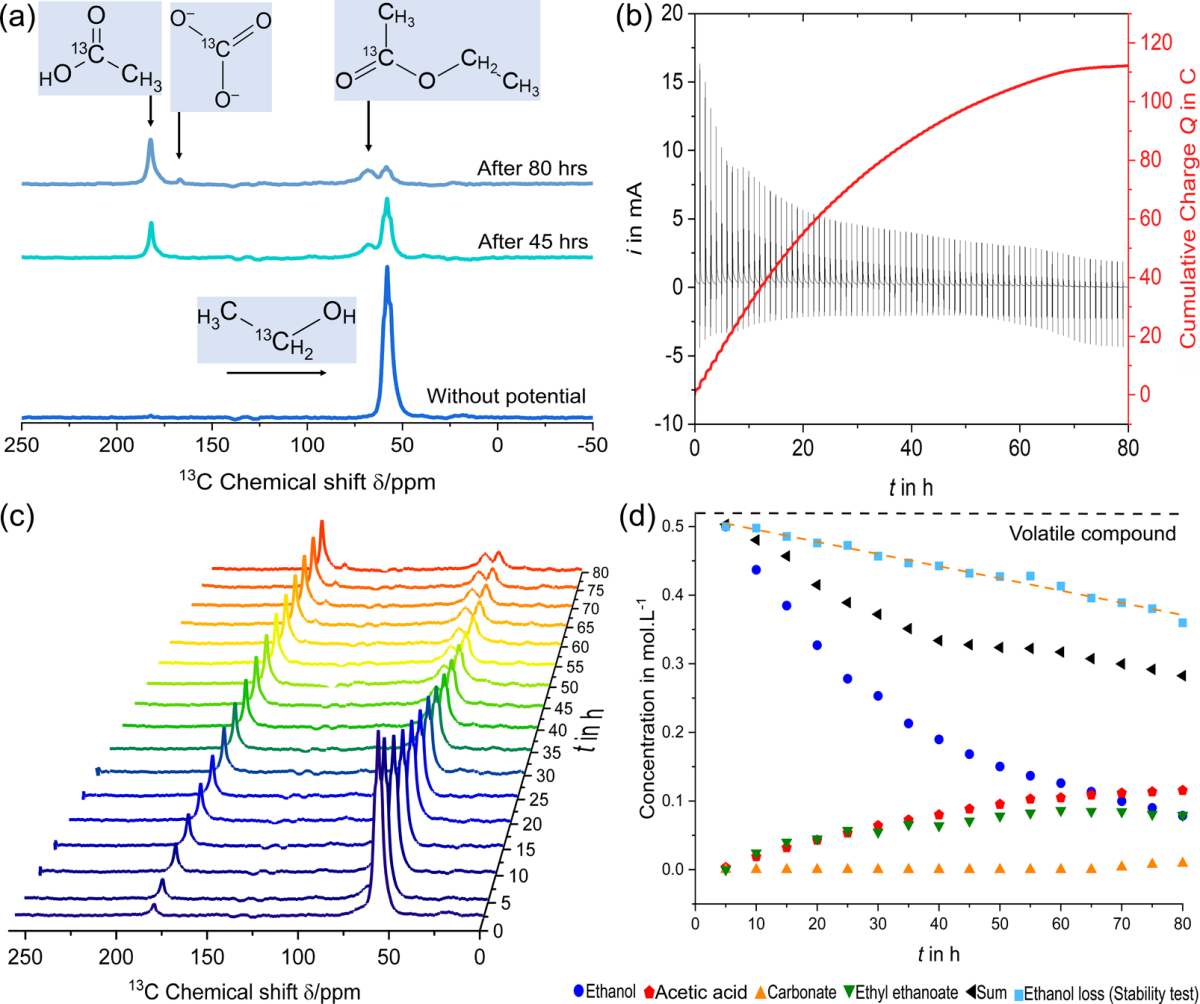
(a) In situ ^13^C SP NMR spectra obtained upon ethanol electrooxidation catalyzed by UiO-66@Pt/Vulcan after 45 and 80 h of reaction with signal assignment; (b) observed current and cumulative charge flow during the in situ oxidation experiment; (c) in situ ^13^C SP NMR spectra collected over 70 h reaction time under −0.1 V vs BPRE; (d) relative concentration of detected species over time, determined from integrated ^13^C NMR signal areas and normalized to the initial ethanol signal. The cumulative relative signal contribution of all detected species is shown with black triangles.

Signal quantification was performed using DMfit software^58^ by integrating the relevant ^13^C resonances. Integrated peak areas of ethanol and product signals were used instead of peak intensities to minimize the influence of line broadening and susceptibility changes during electrochemical operation. The ethanol concentration at each time point was calculated relative to the initial spectrum, which was set as 100%, assuming a linear relationship between NMR signal area and concentration under constant acquisition parameters. As all spectra were obtained using identical pulse conditions and recycle delays, the relative changes in integrated signal areas directly reflected concentration changes over time (Figure 5d).

The ethanol electrooxidation reaction in alkaline electrolyte results in several main products. As ethanol undergoes electrooxidation, it forms acetaldehyde, which subsequently oxidizes to give acetic acid, which partially deprotonates to acetate. This acetate/acetic acid shows a strong signal at 182 ppm that increases over time. A part of acetic acid is eventually oxidized to CO_2_, which reacts with KOH to form carbonate, visible as a signal at 166 ppm, appearing after 60 h of reaction. The carbonate is in chemical equilibrium with carbon dioxide, which is released from the cell, and thus disappears from the observed reaction volume. However, it is not possible to directly detect carbon dioxide in the setup.

In addition to the main products, another significant signal at 68 ppm, corresponding to a side product, namely ethyl ethanoate (ethyl acetate), appeared after 5 h. It is inevitably formed during the electrooxidation reaction from pristine ethanol and the acetic acid produced.^59,60^

Using the Faraday law (eq 1), the theoretical total amount of charge required to oxidize the given ethanol amount to acetic acid completely can be estimated

1 Q=n·z·F

where *Q* is the total charge (in Coulombs), *n* is the amount of reactant (in moles), *z* is the number of electrons exchanged per unit reaction, and *F* is the Faraday constant.

Assuming that only acetate is formed, the oxidation requires a total charge flow of 154 C. By integrating the oxidation current *i* observed in the experiment over time, the cumulative charge flow can be obtained (eq 2)­

2 Q=∫idt

The integral of the current over time offers valuable insights (Figure 5b). After 70 h of oxidation, the total charge flow amounts to 112 C. This suggests that only 72% of the initial alcohol amount could be potentially oxidized. Meanwhile, the NMR data show a consumption of 88% of the initial ethanol amount and 12% residual EtOH. This indicates losses by evaporation of ca. 16% under working conditions. Furthermore, other reaction products are observed, which indicate the occurrence of side reactions, for example, oxidation to acetaldehyde, which requires 2e instead of 4e for acetic acid formation. Lastly, the cell stability experiment demonstrated a loss of ethanol from the setup due to evaporation. Considering all these factors, the cumulative charge flow is in adequate agreement with the NMR data. Since the amounts of evolved CO_2_, evaporated ethanol, and other volatile compounds are not known precisely, the analysis of the Faradaic efficiency of the given catalyst is only possible in a rough approximation. Additionally, the cumulative charge plot (Figure 5b) shows that the catalyst degraded completely after approximately 70 h of oxidation. Although ethanol is clearly still present in the reaction mixture, no further oxidation occurs. The reason behind this degradation could be analyzed by HR-TEM analysis (ESI, Figures S7–S10). The micrographs demonstrate severe sintering of the Pt nanoparticles after catalysis (before catalysis, particle diameter averaged at 2.7 nm, after catalysis >50 nm) as well as restructuring of the MOF into a denser phase, presumably ZrO_2_.

## Conclusion

A newly designed, 3D-printed 3-electrode cell for in situ spectroscopic NMR investigations in parallel with electrocatalytic studies demonstrated significant methodological improvements over earlier work. The polypropylene cell, together with a bipolar reference electrode, facilitates straightforward assembly and better control of the electrochemical environment. The geometric precision of the cell and its mechanical rigidity provide reproducible results during long-term experiments. These advantages were demonstrated using the electrocatalytic ethanol oxidation reaction. This reaction was used here in order to demonstrate the performance of the new cell design compared with the previous one which we have used for ethanol electrocatalytic oxidation studies. However, the developed apparatus can be used for other electrochemical reactions as well. Substrate depletion and reaction product formation were monitored by in situ NMR. This study shows that electrochemical reactions involving volatile compounds and gases can be examined in our three-electrode setup by in situ NMR reliably and cost-effectively. As such, complex potential profiles can be used for the reactions (as was done in this study), and potential-dependent reaction pathways can be studied (as was demonstrated in a recent publication).^47^ These advancements in both methodology and design represent a significant step forward in electrochemical energy production research. While the in situ NMR approach presented here provides direct monitoring of electrochemical reaction pathways under working conditions, it is inherently limited by relatively long acquisition times and sensitivity restrictions. Consequently, the method is well-suited for systems with sufficiently slow kinetics and stable operating conditions and may not be directly applicable to all electrocatalytic systems. Nevertheless, within these limitations, the approach provides unique mechanistic insights that are difficult to access using ex situ or faster operando techniques.

## Supplementary Material



## Notes

PXRD characterization of the catalyst, testing of 3D-printed cell for minimizing leakage, BPRE setup and performance, ^13^C NMR characterization and stability analysis of the 3D-printed cell, electrochemical performance tests, pre and post mortem HRTEM and EDX/STEM analysis of the catalyst (PDF)
