# Supplementary material for: Three-Electrode, 3D-Printed NMR Cells for Electrooxidation Studies
Source: Anal Chem. 2026 Mar 31;98(14):10607–16. doi: 10.1021/acs.analchem.5c07786 (PMC13084627; doi:10.1021/acs.analchem.5c07786)
Supplement: Supplementary file 1 [file ac5c07786_si_001.pdf]

Electronic Supporting Information

for

Three-Electrode 3D-Printed NMR Cells for Electrooxidation Studies

Sara A. Salout [a, §], *Leonid Shupletsov* [b, §], *Irena Senkovska* [b], *Arafat H. Khan* [a], *Stefan Kaskel* [b, *], *Eike Brunner* [a, *]

[a] Chair of Bioanalytical Chemistry, Technische Universität Dresden, Bergstraße 66, 01062 Dresden, Germany

[b] Chair of Inorganic Chemistry I, Technische Universität Dresden, Bergstraße 66, 01062 Dresden, Germany

Table of contents

Figure S1. PXRD characterization of the catalyst……………………………………….………… S3

Figure S2. 3D-printed cell test for minimizing leakage……………………………….…...…… S4

Figure S3. BPRE setup and performance……………………………………………….….… S5

Figure S4. ^{13}C NMR spectroscopy characterization of 3D-printed cell………………………… S6

Figure S5. ^{13}C NMR spectroscopy stability analysis of 3D-printed cell………………………… S6

Figure S6. Electrochemical performance test……………………………………..………. S7

Figure S7. Pre and post mortem HRTEM and EDX/STEM…………………………….……. S8

Figure S8. Pre and mortem EDX/STEM………………………………………………..…… S9

Figure S9. post mortem HRTEM……………………………………………………………… S10

Figure S10. post mortem EDX/STEM………………………………………………………… S10

Reference……………………………………………………………………………………… S11

PXRD characterization of the catalyst

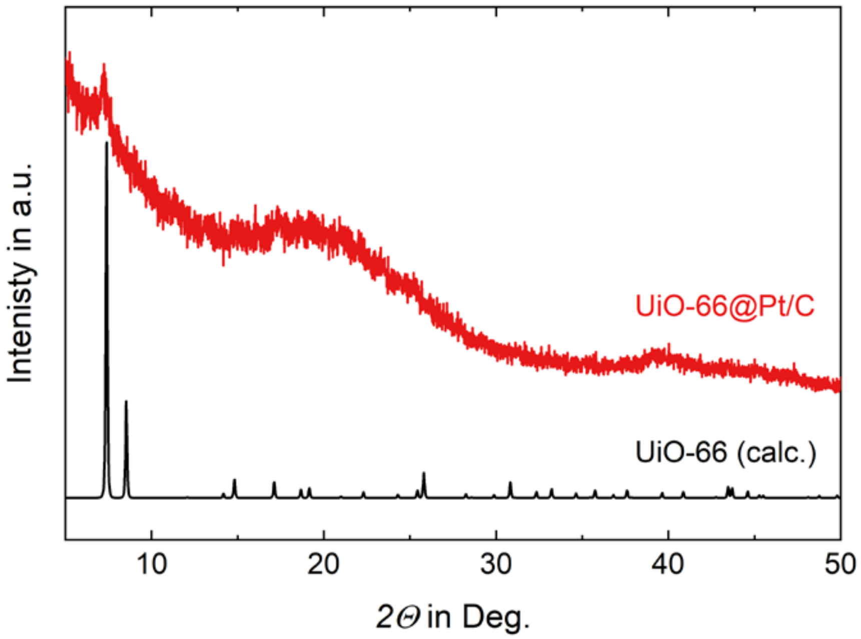

Figure S1. PXRD of the obtained composite materials UiO-66@Pt/C.

3D-printed cell test for minimizing leakage

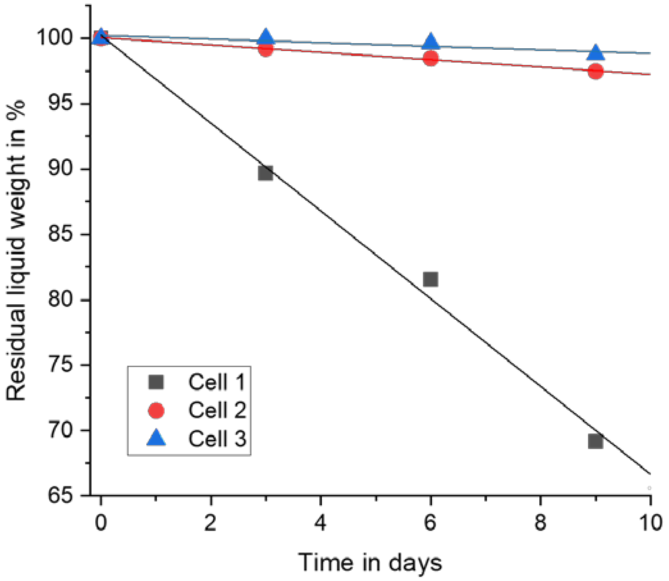

Figure S2. Mass loss of ethanol from three sealed 3D printed cell bodies over 9 days at room temperature.

BPRE setup and performance

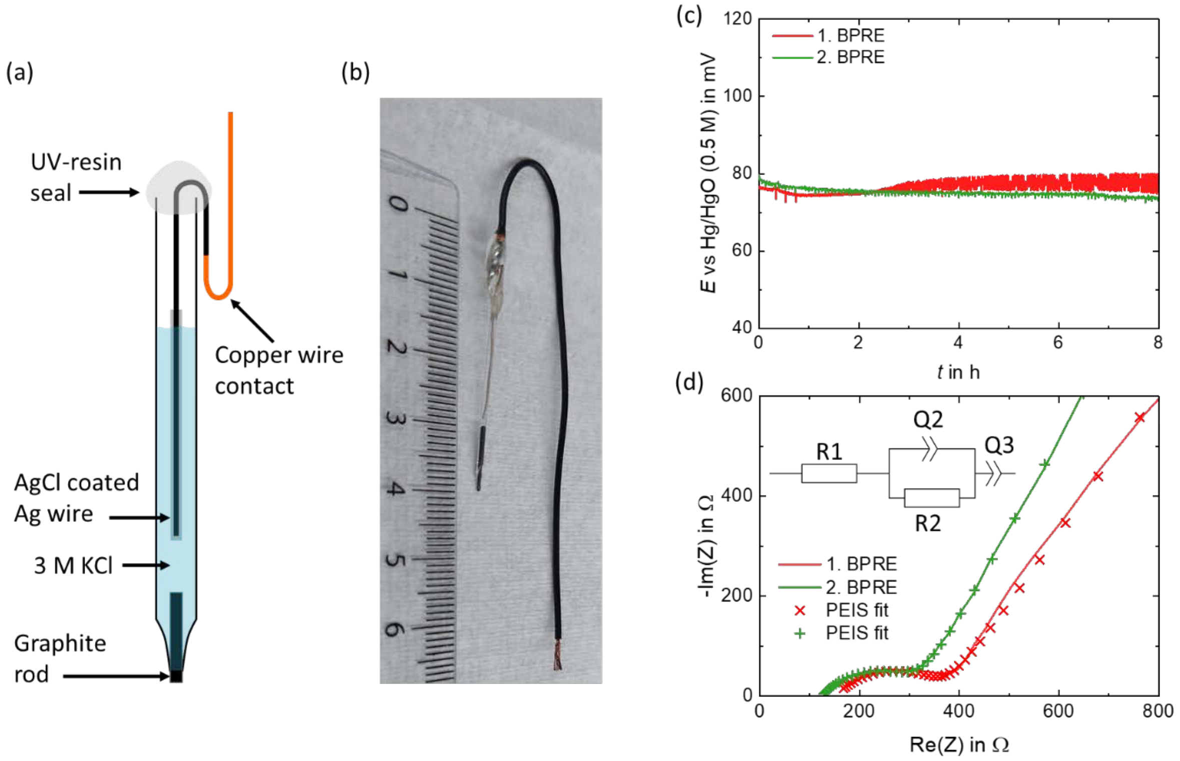

Figure S3. a) Schematic depiction of the leakless, bipolar reference electrode[1], b) Photographical image of manufactured BPRE (scale in cm), c) Open circuit potential (OCV) of two different BPREs *vs*. a commercial Hg/HgO (0.5 mol l^{-1}) reference, d) Potentiostatic Electrochemical Impedance Spectroscopy (PEIS) Nyquist plots of two different BPREs.

^{13}C NMR spectroscopy characterization and stability analysis of 3D-printed cell

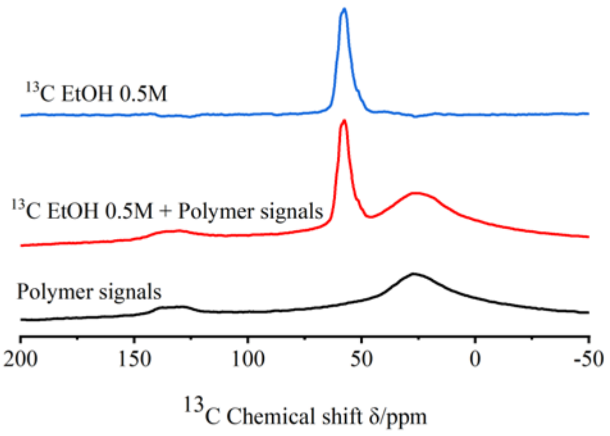

Figure S4. ^{13}C single pulse NMR spectra showing the reference polymer signal of the cell filled with 1 mL of 1 mol l^{-1} KOH (black), the polymer signal of the cell filled with 1 mol l^{-1} ^{13}C-EtOH and 1 mol l^{-1} KOH (1:1 v/v) before (red) and after (blue) subtraction of the polymer signal.

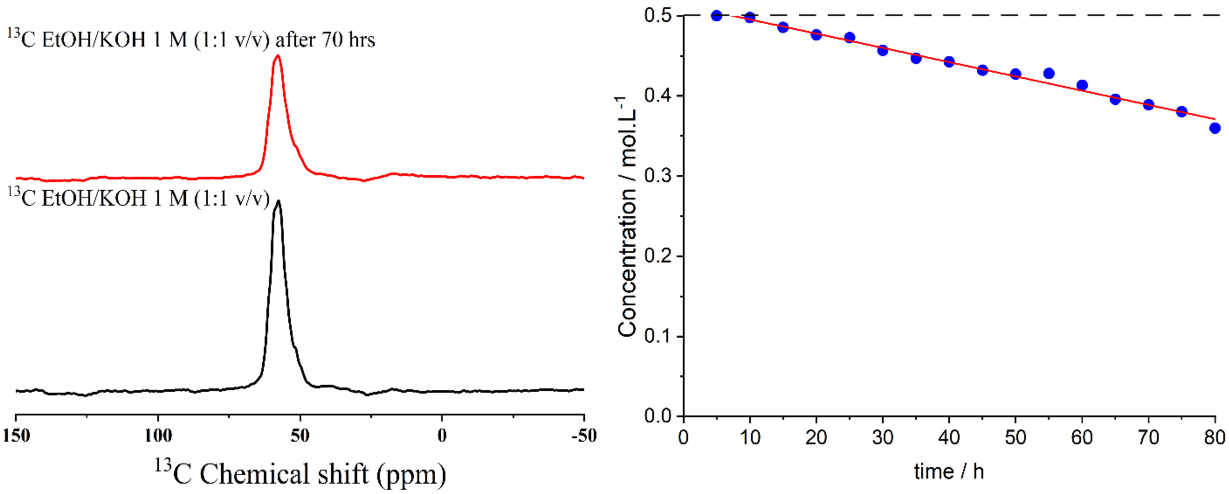

Figure S5. a) ^{13}C single pulse NMR experiment of 1 mol l^{-1} ^{13}C-ethanol and 1 mol l^{-1} KOH (1:1 v/v) before and after 70 h electrooxidation reaction. b) Concentration of EtOH determined from ethanol signal intensity at 58 ppm in the cell stability test over 70 hours.

Electrochemical performance test

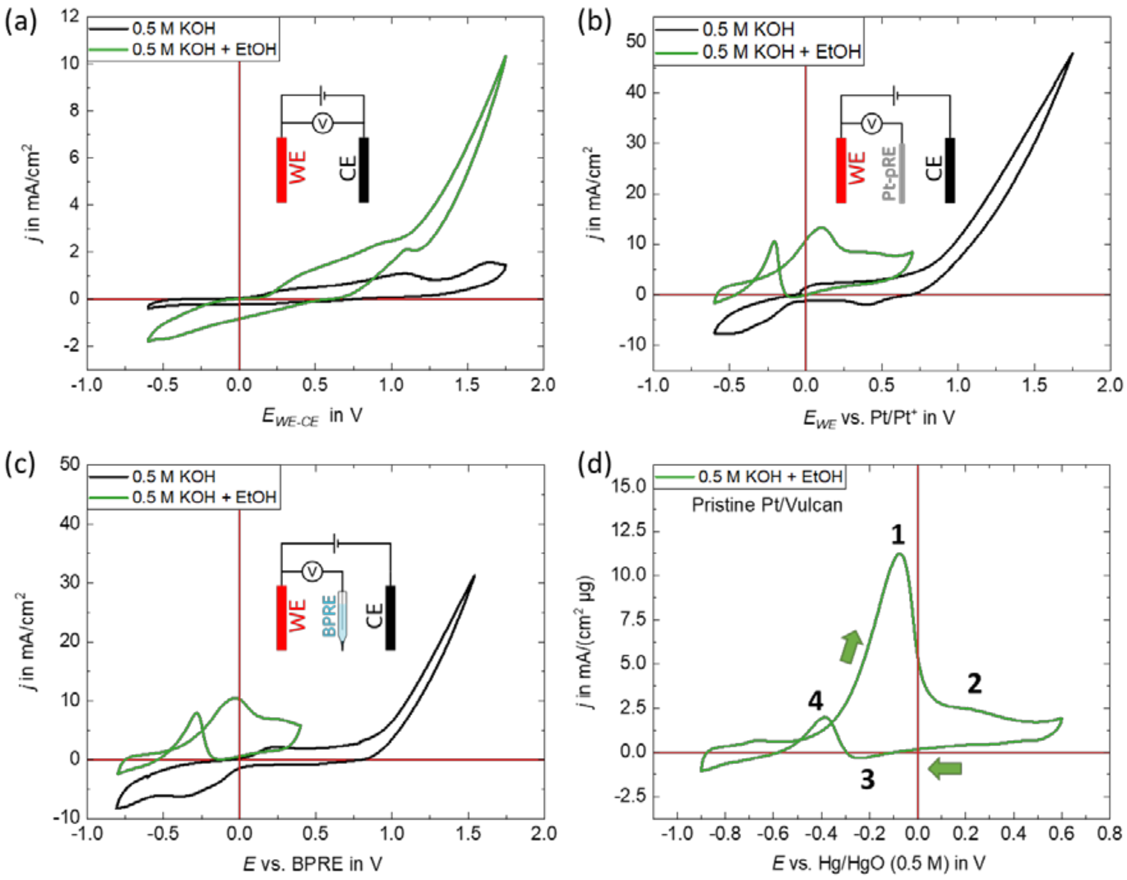

Figure S6. CV collected in the novel poly propylene (PP) cell body, using of UiO-66@Pt/Vulcan as catalyst in ethanol electrooxidation (v = 50 mV s^{-1}) *ex situ* in a) a two-electrode setup; b) a three-electrode setup with a Pt-wire as pseudo reference; (c) a three-electrode setup with a BPRE as reference; d) characteristic CV of ethanol electrooxidation on a Pt/Vulcan catalyst in alkaline medium (arrows mark scan direction), in this case 0.5 mol l^{-1} EtOH in 0.5 mol l^{-1} KOH.

Pre and post mortem HRTEM and EDX/STEM

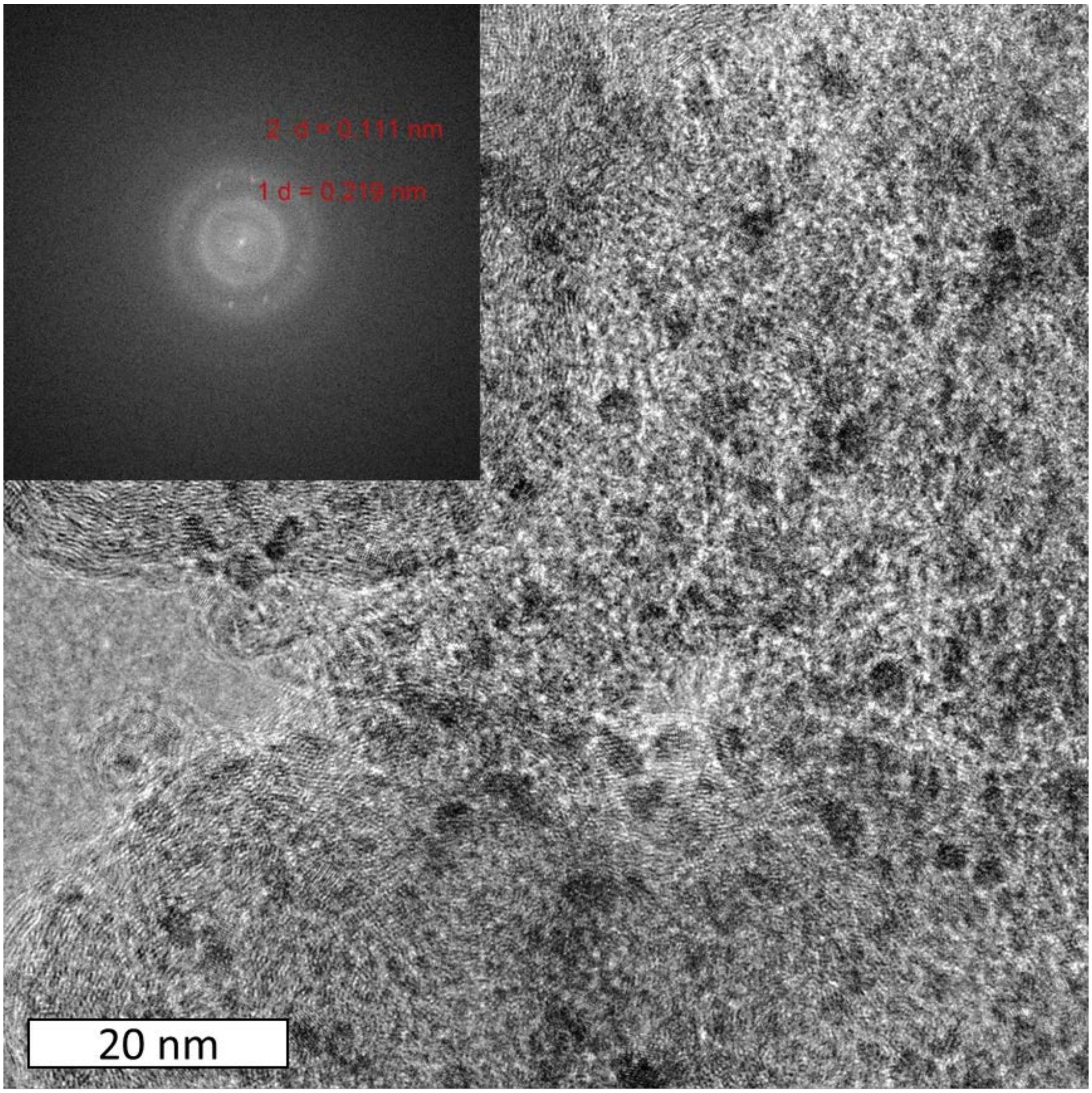

Figures S7. HR-TEM micrograph of the pristine UiO-66@Pt/Vulcan catalyst with Furrier transform in the inset.

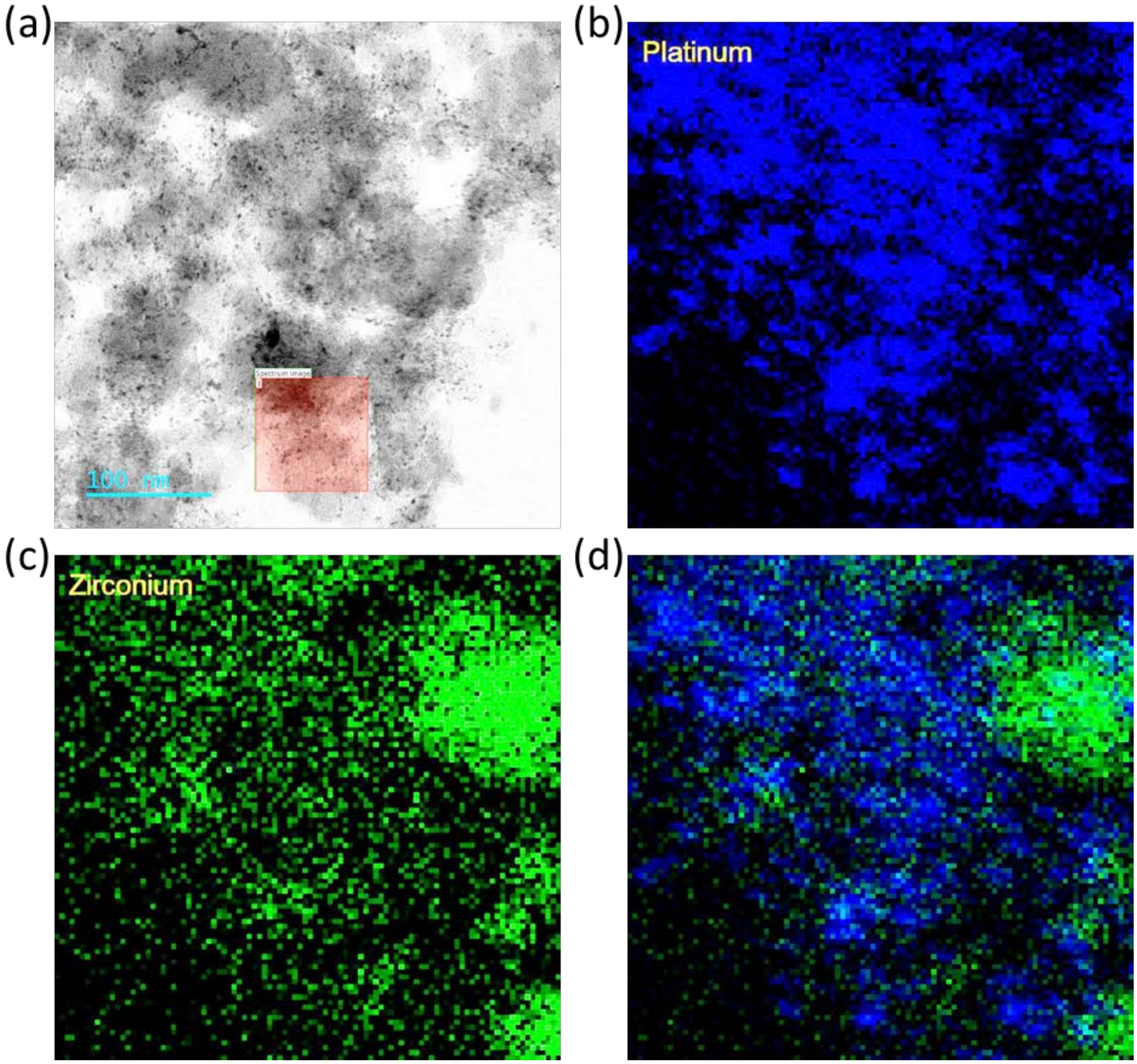

Figure S8. STEM EDX mapping of the UiO-66@Pt/Vulcan catalyst prior to the catalysis: a) Bright-field STEM overview image, b) Distribution of platinum, c) Distribution of zirconium, d) Mixed color map of platinum and zirconium.

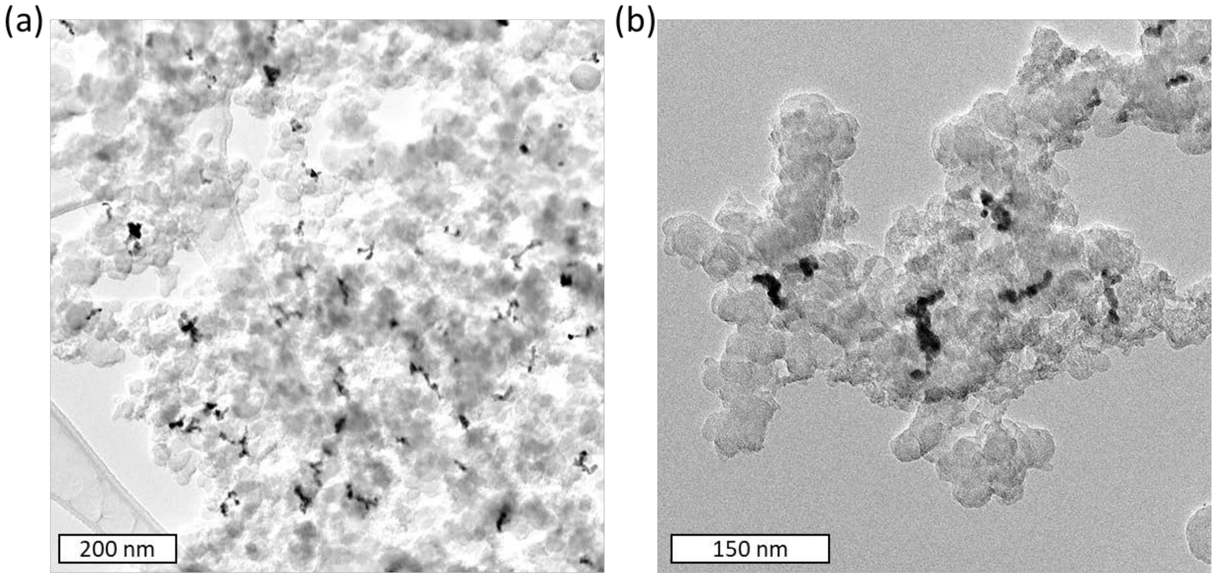

Figure S9. HR-TEM micrographs of the UiO-66@Pt/Vulcan catalyst after catalysis.

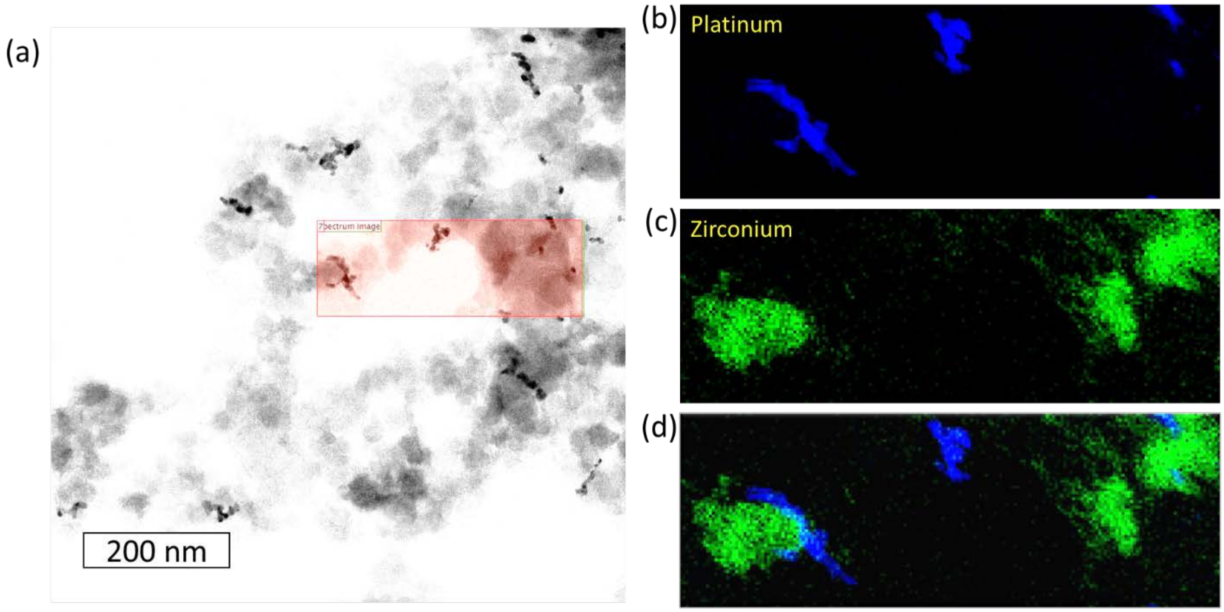

Figure S10. STEM EDX mapping of the UiO-66@Pt/Vulcan after catalysis: a) Bright-field STEM overview image, b) Distribution of platinum, c) Distribution of zirconium, d) Mixed color map of platinum and zirconium.

References

(1) Walker, N. L.; Dick, J. E. Leakless, bipolar reference electrodes: fabrication, performance, and miniaturization. *Anal. Chem.* **2021**, *93* (29), 10065–10074.
